# Supplementary material for: Probiotics Attenuate Food Allergy via Short-Chain Fatty Acids-Mediated Immune Modulation and Gut Barrier Restoration
Source: Foods. 2025 Nov 18;14(22):3953. doi: 10.3390/foods14223953 (PMC12652458; doi:10.3390/foods14223953)
Supplement: Supplementary file 1 [file foods-14-03953-s001.zip › Supplementary Figure S1 and Table S1.pdf]

Supplementary Figure S1: Schematic experimental illustration of the methods of the mouse food allergy model

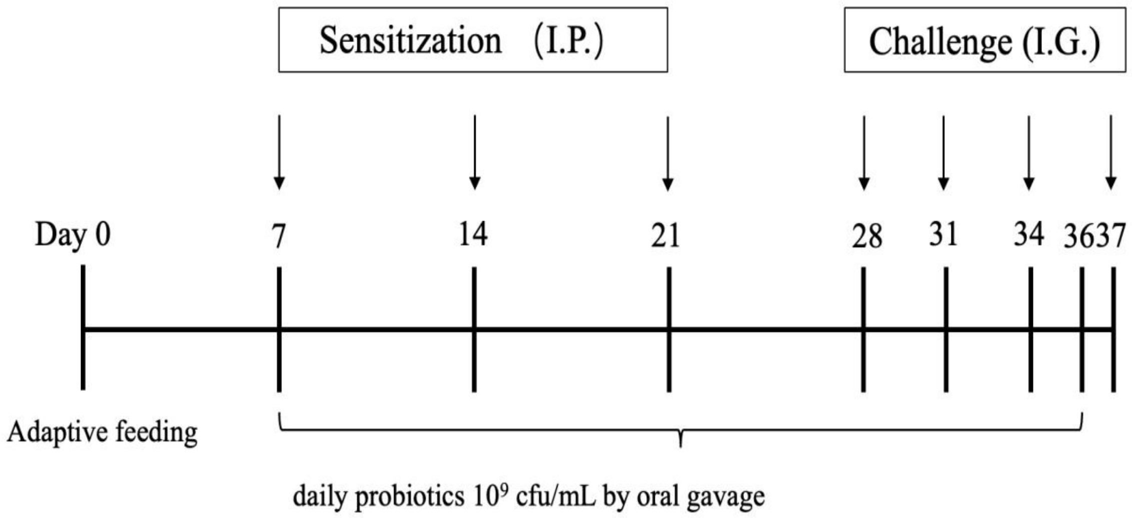

Supplementary Table S1: Table illustrating mouse allergy symptom score

Score	Symptom	Diarrhea
0	No obvious symptoms	No obvious symptoms
1	Scratch the ears and nose	Feces are in granular form with mucus
2	Swelling of ears and eyes, rapid breathing	Fecal loss of basic particle structure
3	Red rashes on the mouth and tail, difficulty breathing, and asthma	Irregular stool shape with a large amount of yellow urine
4	Body twitching, muscle contraction, slow or stopped behavior	Irregular shaped liquid feces with a small amount of fecal residue in the anal area
5	Shock, death;	Severe watery stool with clumping in the anal area
