# Supplementary material for: Probiotics Attenuate Food Allergy via Short-Chain Fatty Acids-Mediated Immune Modulation and Gut Barrier Restoration
Source: Foods. 2025 Nov 18;14(22):3953. doi: 10.3390/foods14223953 (PMC12652458; doi:10.3390/foods14223953)
Supplement: Supplementary file 1 [file foods-14-03953-s001.zip › Supplementary Table S2.pdf]

Supplementary Table S2: Spearman correlation (R-value) between gut microbiota, short-chain fatty acids and specific allergen factors

	**Spearman correlation (R-value) between gut microbiota, short-chain fatty acids and specific-allergen factors**																		
Taxons	Histamine	MCP-1	IgE	IgG	IgG1	IL-2	IL-4	IFN-γ	TGF-β1	IL-5	TNF-α	CD4 CD25	ZO-1	Claudin-1	Acetic Acid	Butyric Ac	Isobutyric	Pentanoic	Propionic
Muribaculaceae	-0.6	-0.25714	-0.6	-0.65714	-0.37143	-0.25714	-0.71429	-0.14286	0.085714	-0.71429	-0.65714	0.257143	0.428571	0.2	0.085714	0.2	0.028571	0.2	0.428571
Lachnospiraceae_NK4A136_gro	-0.31429	-0.25714	-0.02857	-0.37143	-0.37143	-0.25714	-0.14286	0.257143	0.942857	0.142857	-0.37143	0.542857	0.428571	0.771429	0.828571	0.657143	0.657143	0.657143	0.714286
Alistipes	0.028571	-0.31429	-0.54286	-0.77143	-0.48571	-0.31429	-0.71429	-0.02857	0.485714	-0.14286	-0.77143	0.885714	0.714286	0.942857	0.657143	0.542857	0.6	0.542857	0.714286
Alloprevotella	0.714286	-0.37143	-0.48571	-0.31429	-0.31429	-0.37143	-0.42857	-0.6	-0.42857	-0.14286	-0.31429	0.542857	0.428571	0.314286	-0.37143	-0.42857	-0.31429	-0.42857	-0.31429
Helicobacter	0.314286	0.6	0.6	0.942857	0.771429	0.6	0.771429	0.2	-0.2	0.6	0.942857	-0.54286	-0.82857	-0.48571	0.028571	0.085714	0.085714	0.085714	-0.2
Bacteroides	0.085714	-0.54286	-0.42857	0.085714	-0.25714	-0.54286	-0.14286	-0.88571	-0.14286	-0.42857	0.085714	0.257143	0.142857	0.028571	-0.42857	-0.37143	-0.6	-0.37143	-0.25714
Clostridia_vadinBB60_group	0.142857	0.542857	0.428571	0.942857	0.771429	0.542857	0.657143	-0.02857	-0.48571	0.257143	0.942857	-0.71429	-0.88571	-0.77143	-0.31429	-0.14286	-0.25714	-0.14286	-0.37143
Clostridia_UCG-014	-0.14286	0.6	0.714286	0.771429	0.657143	0.6	0.771429	0.428571	-0.37143	0.314286	0.771429	-1	-0.82857	-0.94286	-0.37143	-0.25714	-0.25714	-0.25714	-0.48571
Prevotellaceae_UCG-001	-0.94286	-0.2	-0.08571	-0.25714	-0.25714	-0.2	-0.14286	0.142857	0.542857	-0.42857	-0.25714	-0.08571	0.142857	0.028571	0.257143	0.314286	0.085714	0.314286	0.428571
Odoribacter	0.771429	-0.02857	-0.25714	-0.37143	-0.14286	-0.02857	-0.37143	-0.14286	-0.65714	0.028571	-0.37143	0.257143	0.314286	0.085714	-0.42857	-0.48571	-0.2	-0.48571	-0.42857
Muribaculum	-0.02857	0.314286	-0.37143	-0.14286	0.257143	0.314286	-0.42857	-0.08571	-0.71429	-0.42857	-0.14286	-0.2	-0.14286	-0.37143	-0.31429	-0.08571	-0.14286	-0.08571	-0.02857
Lachnospiraceae_UCG-001	-0.25714	-0.88571	-0.54286	-0.37143	-0.71429	-0.88571	-0.37143	-0.77143	0.428571	-0.54286	-0.37143	0.6	0.6	0.485714	-0.08571	-0.14286	-0.37143	-0.14286	0.085714
Turicibacter	0.028571	0.542857	-0.14286	0.314286	0.6	0.542857	-0.08571	-0.08571	-0.82857	-0.25714	0.314286	-0.48571	-0.54286	-0.65714	-0.37143	-0.08571	-0.2	-0.08571	-0.14286
Roseburia	0.371429	0.028571	0.485714	0.6	0.2	0.028571	0.657143	-0.02857	0.142857	0.542857	0.6	-0.14286	-0.31429	-0.08571	-0.02857	-0.14286	-0.08571	-0.14286	-0.31429
Colidextribacter	0.542857	-0.02857	-0.25714	-0.6	-0.25714	-0.02857	-0.48571	0.2	0.142857	0.257143	-0.6	0.714286	0.542857	0.771429	0.485714	0.314286	0.6	0.314286	0.371429
Parabacteroides	-0.71429	-0.31429	-0.42857	0.085714	-0.08571	-0.31429	-0.2	-0.54286	0.371429	-0.6	0.085714	0.142857	-0.02857	0.085714	0.2	0.371429	-0.08571	0.371429	0.485714
Oscillibacter	-0.25714	-0.88571	-0.54286	-0.37143	-0.71429	-0.88571	-0.37143	-0.77143	0.428571	-0.54286	-0.37143	0.6	0.6	0.485714	-0.08571	-0.14286	-0.37143	-0.14286	0.085714
Rikenellaceae_RC9_gut_group	0.085714	-0.65714	-0.31429	-0.14286	-0.48571	-0.65714	-0.14286	-0.6	0.428571	-0.14286	-0.14286	0.6	0.428571	0.542857	0.085714	-0.02857	-0.14286	-0.02857	0.085714
Rikenella	-0.14286	-0.08571	-0.42857	-0.6	-0.25714	-0.08571	-0.6	0.142857	0.542857	-0.08571	-0.6	0.714286	0.485714	0.828571	0.828571	0.771429	0.771429	0.771429	0.885714
Lachnoclostridium	-0.02857	-0.6	-0.6	-0.37143	-0.48571	-0.6	-0.48571	-0.6	0.485714	-0.31429	-0.37143	0.828571	0.542857	0.771429	0.371429	0.314286	0.142857	0.314286	0.485714
Lactobacillus	-0.77143	-0.37143	0.085714	-0.02857	-0.31429	-0.37143	0.142857	0.028571	0.885714	-0.14286	-0.02857	0.085714	0.142857	0.257143	0.428571	0.371429	0.142857	0.371429	0.428571
Blautia	-0.65714	-0.08571	0.371429	0.257143	-0.02857	-0.08571	0.428571	0.257143	0.828571	0.142857	0.257143	-0.14286	-0.14286	0.085714	0.485714	0.428571	0.257143	0.428571	0.371429
[Eubacterium]_siraeum_group	0.142857	-0.48571	0.085714	-0.42857	-0.6	-0.48571	-0.02857	-0.02857	-0.14286	-0.08571	-0.42857	-0.02857	0.485714	-0.08571	-0.65714	-0.82857	-0.6	-0.82857	-0.71429
Lachnospiraceae_FCS020_grou	0.142857	0.142857	0.371429	0.6	0.314286	0.142857	0.542857	0.028571	0.371429	0.485714	0.6	-0.02857	-0.37143	0.085714	0.371429	0.314286	0.257143	0.314286	0.142857
ASF356	-0.02857	0.085714	-0.14286	-0.6	-0.2	0.085714	-0.42857	0.485714	0.428571	0.142857	-0.6	0.485714	0.428571	0.657143	0.714286	0.6	0.771429	0.6	0.657143
GCA-900066575	-0.31429	0.428571	-0.25714	0.428571	0.6	0.428571	-0.08571	-0.2	-0.14286	-0.25714	0.428571	-0.14286	-0.54286	-0.2	0.314286	0.6	0.257143	0.6	0.542857
[Eubacterium]_xylanophilum_g	0.657143	-0.14286	-0.37143	-0.02857	-0.02857	-0.14286	-0.25714	-0.6	-0.77143	-0.2	-0.02857	0.142857	0.085714	-0.14286	-0.65714	-0.6	-0.54286	-0.6	-0.54286
Acetatifactor	1	0.257143	0.142857	0.085714	0.2	0.257143	0.085714	0.085714	-0.48571	0.542857	0.085714	0.142857	-0.02857	0.085714	-0.14286	-0.25714	0.085714	-0.25714	-0.37143
Monoglobus	-0.31429	-0.02857	-0.14286	-0.65714	-0.31429	-0.02857	-0.42857	0.428571	0.257143	-0.14286	-0.65714	0.2	0.428571	0.314286	0.314286	0.257143	0.371429	0.257143	0.371429
Bilophila	-0.2	-0.71429	-0.48571	-0.25714	-0.54286	-0.71429	-0.31429	-0.65714	0.542857	-0.37143	-0.25714	0.657143	0.485714	0.6	0.2	0.142857	-0.08571	0.142857	0.314286
Marvinbryantia	0.314286	0.6	0.714286	0.2	0.371429	0.6	0.485714	0.942857	0.257143	0.885714	0.2	-0.2	-0.25714	0.085714	0.542857	0.371429	0.714286	0.371429	0.142857
Lachnospiraceae_UCG-006	-0.6	-0.82857	-0.6	-0.48571	-0.71429	-0.82857	-0.48571	-0.71429	0.142857	-0.88571	-0.48571	0.257143	0.542857	0.085714	-0.42857	-0.37143	-0.65714	-0.37143	-0.08571
[Eubacterium]_coprostanoliger	0.314286	-0.14286	0.085714	0.085714	-0.08571	-0.14286	0.142857	-0.02857	0.542857	0.428571	0.085714	0.485714	0.142857	0.6	0.542857	0.371429	0.428571	0.371429	0.314286
Tuzzerella	-0.31429	-0.2	-0.42857	0.257143	0.085714	-0.2	-0.14286	-0.65714	0.142857	-0.42857	0.257143	0.2	-0.14286	0.085714	0.142857	0.314286	-0.08571	0.314286	0.371429
UCG-003	-0.6	-0.02857	-0.02857	-0.37143	-0.2	-0.02857	-0.2	0.428571	0.771429	-0.02857	-0.37143	0.257143	0.257143	0.485714	0.771429	0.714286	0.657143	0.714286	0.771429
Desulfovibrio	0.6	0.828571	0.6	0.485714	0.714286	0.828571	0.485714	0.714286	-0.14286	0.885714	0.485714	-0.25714	-0.54286	-0.08571	0.428571	0.371429	0.657143	0.371429	0.085714
UCG-010	-0.08571	0.885714	0.314286	0.714286	0.942857	0.885714	0.371429	0.371429	-0.31429	0.257143	0.714286	-0.6	-0.88571	-0.54286	0.257143	0.485714	0.371429	0.485714	0.257143
Anaeroplasma	-0.77143	-0.14286	-0.48571	0.028571	0.028571	-0.14286	-0.31429	-0.42857	0.257143	-0.65714	0.028571	0.085714	-0.08571	0.028571	0.257143	0.485714	0.028571	0.485714	0.6
Butyricicoccus	-0.25714	0.771429	0.428571	0.828571	0.885714	0.771429	0.542857	0.314286	-0.25714	0.2	0.828571	-0.77143	-0.94286	-0.71429	0.085714	0.314286	0.142857	0.314286	0.085714
Escherichia-Shigella	0.485714	0.085714	0.2	0.6	0.314286	0.085714	0.428571	-0.37143	-0.6	0.142857	0.6	-0.37143	-0.42857	-0.54286	-0.65714	-0.6	-0.6	-0.6	-0.71429
Tyzzerella	0.257143	-0.14286	0.314286	-0.14286	-0.25714	-0.14286	0.2	0.371429	0.714286	0.6	-0.14286	0.428571	0.314286	0.657143	0.6	0.314286	0.542857	0.314286	0.257143
Ruminococcus	0.542857	0.257143	0.257143	0.714286	0.485714	0.257143	0.485714	-0.25714	-0.48571	0.314286	0.714286	-0.31429	-0.54286	-0.42857	-0.37143	-0.31429	-0.31429	-0.31429	-0.48571
Anaerotruncus	-0.08571	0.085714	0.085714	-0.02857	0.028571	0.085714	0.028571	0.314286	0.771429	0.371429	-0.02857	0.428571	0.085714	0.657143	0.942857	0.828571	0.828571	0.828571	0.771429
UBA1819	-0.42857	0.028571	0.028571	0.371429	0.2	0.028571	0.2	-0.08571	0.6	0.028571	0.371429	0.085714	-0.25714	0.2	0.6	0.657143	0.371429	0.657143	0.6
Romboutsia	0.314286	0.6	0.028571	0.428571	0.657143	0.6	0.085714	-0.02857	-0.94286	-0.02857	0.428571	-0.54286	-0.6	-0.71429	-0.48571	-0.25714	-0.25714	-0.25714	-0.37143
UCG-009	0.2	0.428571	-0.02857	-0.25714	0.2	0.428571	-0.25714	0.542857	0.257143	0.371429	-0.25714	0.371429	0.085714	0.542857	0.828571	0.771429	0.942857	0.771429	0.714286
Enterorhabdus	0.485714	0.028571	0.142857	0.314286	0.142857	0.028571	0.257143	-0.08571	0.257143	0.485714	0.314286	0.314286	-0.08571	0.371429	0.371429	0.257143	0.314286	0.257143	0.142857
